# Harnessing monocyte dynamics for treatment of multiple sclerosis; insights from experimental model studies

**DOI:** 10.1093/immadv/ltaf003

**Published:** 2025-04-20

**Authors:** Aqsa Bibi, Zhenjiang Yu, Lv Cui, Guiwen Yang

**Affiliations:** Shandong Provincial Key Laboratory of Animal Resistance Biology, College of Life Sciences, Shandong Normal University, Jinan 250061, China; Shandong Provincial Key Laboratory of Animal Resistance Biology, College of Life Sciences, Shandong Normal University, Jinan 250061, China; Medical Science and Technology Innovation Center, Shandong First Medical University & Shandong Academy of Medical Sciences, Jinan, Shandong 250117, China; Shandong Provincial Key Laboratory of Animal Resistance Biology, College of Life Sciences, Shandong Normal University, Jinan 250061, China

**Keywords:** monocytes, multiple sclerosis, Ly6C, patrolling, innate immune system

## Abstract

Monocytes are central to the innate immune system’s response to infection or injury. In murine, these cells are classified into distinct subsets: classical monocytes, defined by elevated Ly6C expression (Ly6C^hi^), intermediate monocytes (Ly6C^int^), and non-classical inflammatory monocytes, characterized by low Ly6C expression (Ly6C^low^). Monocytes recruited to tissues differentiate into macrophages, which can be pro-inflammatory or anti-inflammatory, thereby influencing disease processes and outcomes. The principal function of classical monocytes is the mediation of pro-inflammatory reactions, whereas non-classical monocytes are associated with repair and anti-inflammatory processes, patrolling the lumen of the vessels. Growing evidence highlights the importance of monocytes in multiple sclerosis (MS), an autoimmune and neurodegenerative disease of the central nervous system (CNS). Recent studies indicate that modulation of the innate immune system, focusing specifically on the shift from Ly6C^hi^ to Ly6C^low^ monocytes, is an effective therapeutic strategy for neurodegenerative diseases, such as Alzheimer’s and MS. This transition is crucial for switching the immune response from inflammation to tissue repair and inflammation resolution, emphasizing the plasticity of monocytes and their potential as targets in MS. This review differs from prior studies in that it focuses solely on animal models of MS, which either directly perturb or study monocytes, or where therapeutic approaches mediate their protective effects through monocytes. Such details permit a subtle comprehension of monocyte dynamics in the context of MS.

## Graphical Abstract

**Graphical Abstract:**
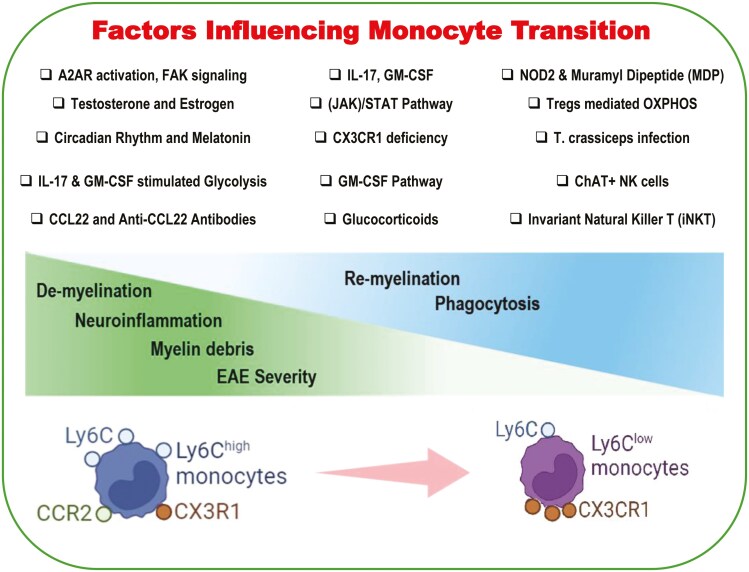

## Introduction

Monocytes, a type of phagocytic cells in the blood, play a crucial role in immune surveillance, inflammation, and tissue repair. These cells of the innate immune system originate from hematopoietic stem cells (HSCs) in the bone marrow and are characterized for their plasticity and ability to differentiate into a variety of cell types, including macrophages and dendritic cells, once they migrate to other tissues. During early development, monocytes arise from HSCs in the bone marrow through a well-defined lineage pathway. The first differentiation of HSCs involves multipotent progenitors (MPPs), which in turn contribute to the formation of common myeloid progenitors (CMPs) and further into granulocyte-macrophage progenitors (GMPs), which are the precursors of monocytes [1]. In adults, this is a continuous process, and adult HSCs in the bone marrow give rise to monocytes throughout their life [2], which are regulated by several factors, such as colony-stimulating factor 1 (CSF-1), interleukin-3 (IL-3), and granulocyte-macrophage colony-stimulating factor (GM-CSF) [3].

After maturation, monocytes exit the bone marrow, enter the bloodstream, and circulate until recruited to sites of infection or injury. They are guided to tissues by chemokine gradients, adhesion molecules, and signals from endothelial cells. In the brain, monocytes have the ability to cross the blood–brain barrier (BBB) under pathological conditions. Once monocytes infiltrate tissues, they differentiate into macrophages that adopt either pro-inflammatory or anti-inflammatory phenotypes, contributing to tissue inflammation, repair, or homeostasis depending on the context. In the central nervous system (CNS), these infiltrating monocytes can differentiate into macrophages, although they also function in immunity, debris clearance, and tissue repair. Under conditions, such as MS, monocytes can worsen CNS inflammation and demyelination but also participate in repair and remyelination processes [4].

Monocytes exhibit both conserved and species-specific characteristics, particularly between humans and rodents, which influence their identification, functional interpretation, and translational research ramifications. Based on the expression of specific markers, monocytes are categorized into three subsets: classical monocytes in humans are defined by their high levels of CD14 and low levels of CD16 (CD14^++^CD16^-^), and in mice by high expression of Ly6C (Ly6C^hi^) and CCR2 (CCR2^++^). Such monocytes are linked to pro-inflammatory reactions and have anti-inflammatory capabilities that are critical in terminating inflammation and initiating tissue repair mechanisms. They can be converted to macrophages with an M2-like phenotype, which is associated with healing and tissue regeneration. In humans, intermediate monocytes are identified by CD14^++^CD16^+^ expression, a demarcation that has not yet been well established in humans and mice. In mice, monocyte functions in antigen presentation and inflammatory cytokine production are presumed to be fulfilled by populations of monocytes with similar activities. In humans, non-classical monocytes are identified through the expression of CD14^+^CD16^++,^ and in mice, low expression of Ly6C (Ly6C^low^), high levels of CX3CR1, and low expression of CCR2. These monocytes patrol the endothelial lining of blood vessels, responding rapidly to viral infections and tissue injury [5, 6].

The circulating lifespan of monocytes is relatively short, ranging from several hours to a few days, allowing for quick turnover. However, upon migrating into tissues and undergoing differentiation, their lifespan is significantly extended, depending on the tissue environment and the state of inflammation. The transition from classical (inflammatory) to patrolling (non-classical) monocyte subsets has recently emerged as a pivotal factor in multiple sclerosis (MS) treatment, despite their involvement in MS pathology, which has been documented over the past few decades. Consequently, it is now possible to uncover the complex dynamics that shape the pathophysiology of the disease. In this study, we aimed to review the available literature on the influence of monocytes on the development, progression, and severity of an animal model of MS, which has been summarized in **Table 1**. In addition, different monocyte functional pathways in MS models and drugs that alleviate MS through monocytes have also been described.

**Table 1.** Monocyte studies in animal models of MS

Study focus	Key findings	Implications for MS therapy	References
NOD2 and muramyl dipeptide (MDP)	MDP treatment delayed onset of EAE, shifted toward Ly6C^^low^ monocytes.	Suggests targeting monocyte subset shifts as a therapeutic strategy.	[7].
CCL22 and anti-CCL22 antibodies	Anti-CCL22 delayed EAE onset, reduced severity, highlighting CCL22’s role.	Highlights potential of CCL22 neutralization in early stages of MS.	[8, 9]
Invariant natural killer T (iNKT) cells	Activation of iNKT cells led to a decrease in Ly6C^hi monocytes and increased M2 macrophages.	Supports modulating iNKT cell activity to influence monocyte function.	[10]
Granulocyte-macrophage colony-stimulating factor (GM-CSF)	GM-CSF-dependent pathway mobilized proinflammatory monocytes before EAE relapses.	Points to GM-CSF signaling as a viable target for MS intervention.	[11]
Janus kinase (JAK)/STAT pathway	JAK inhibition reduced monocyte-derived dendritic cell activities and EAE severity.	Indicates JAK/STAT pathway as a promising target for MS treatment.	[12, 13]
CX3CR1 in monocytes	CX3CR1 deficiency aggravated EAE symptoms, pointing to its regulatory role.	Emphasizes the importance of CX3CR1 in regulating neuroinflammation.	[14]
IL-10-producing regulatory B cells (Bregs)	Breg transfusion reversed EAE symptoms, highlighting their therapeutic potential.	Illustrates the potential of Bregs in normalizing the CNS immune environment.	[15]
Acetylcholine-producing NK Cells	ChAT+ NK cells mitigated CNS damage and regulated monocyte/macrophage activity.	Suggests leveraging ChAT+ NK cells for immune modulation in MS.	[16]
Monocytes and their derived APCs	Identified monocyte subsets with distinct roles in EAE, highlighting therapeutic potential.	Highlights the importance of targeting specific monocyte subsets.	[17, 18]
Maturation of circulating classical monocytes	OM-MOG induced maturation of monocytes to a less inflammatory state.	Advocates for inducing peripheral monocyte maturation as a therapeutic approach.	[19]
Circadian rhythm and melatonin	Circadian rhythms influenced monocyte functionality, affecting MS progression.	Indicates the potential impact of circadian rhythm on treatment efficacy.	[20]
Alternatively activated macrophages (AAMs)	*T. crassiceps* infection induced differentiation of monocytes into AAMs, mitigating EAE severity.	Demonstrates the therapeutic potential of helminth-induced immunomodulation.	[21]
Kinetics of classical monocytes	Modulation of CD11b+Ly6C+ cell kinetics affected EAE progression, emphasizing their role.	Highlights the importance of understanding monocyte kinetics for therapy.	[22–24]

## NOD2

One of the most significant roles of nucleotide-binding oligomerization domain-containing protein 2 (NOD2) is to act as an innate immune system receptor that recognizes microbial motifs. A study by Fani Maleki and colleagues investigated the effects of Muramyl Dipeptide (MDP), a NOD2 receptor agonist, in two mouse models of MS: cuprizone and experimental autoimmune encephalomyelitis (EAE) models. The study addressed the effects of MDP on different monocyte subsets and assessed whether MDP could be used as a treatment for MS. MDP treatment delayed the onset of EAE and reduced leukocyte infiltration into the CNS prior to disease onset. Most importantly, a sharp increase in the Ly6C^low^ monocyte ratio to Ly6C^hi^ was observed in MDP-treated mice, which was associated with an enhancement in the resistance of the mice to the development of EAE. In the cuprizone model, MDP treatment promoted the conversion of Ly6C^hi^ to Ly6C^low^ monocytes and minimized the extent of demyelination. This implies that MDP modulates monocyte subsets reduces brain inflammation and may provide a basis for proper remyelination by efficient phagocytosis of myelin debris in both the EAE and cuprizone models [7].

## CCL22

Macrophage-derived chemokines (MDC; CCL22) are cytokines secreted by dendritic cells and macrophages that influence the immune system by predominantly directing T cells and dendritic cells toward inflamed sites or lymphoid tissues. CCL22 exerts its functions by binding to CCR4, which is found on the surface of certain T cell subsets (particularly Th2 cells) and monocytes. Dogan *et al*. were the first to explore the role of CCL22 in the pathogenesis of EAE and discovered that its expression in draining lymph nodes and the spinal cord correlated with disease development and severity. Administration of anti-CCL22 antibodies at the time of autoantigen immunization prolonged the onset of clinical symptoms, reduced the severity of both initial and recurrent relapses, and was associated with decreased pathology and leukocyte influx into the CNS, particularly of activated CD11b+Ly6C^hi^ macrophages. Nonetheless, in disease onset, treatment with anti-CCL22 antibodies did not influence progression, highlighting a critical role for CCL22 in disease induction rather than its progression. These findings underscore the importance of CCL22 in EAE pathogenesis by mediating macrophage chemoattraction and effector function, posing a potential therapeutic target for MS. It is noteworthy that CCL22 neutralization shifts the macrophages to a less inflammatory phenotype, indicating its involvement in EAE development through macrophage chemoattraction and effector function modulation [8].

Furthermore, in the EAE model, Forde *et al*. investigated the role of the chemokine receptor CCR4, showing increased levels of CCR4 and its ligand CCL22 in both the periphery and CNS during EAE. CCR4-deficient mice (CCR4^−/−^) exhibited delayed EAE onset, reduced disease incidence, and decreased severity compared to wild-type mice. Notably, this attenuation in disease severity was not attributed to altered peripheral T-cell responses, which remained unaffected in CCR4^−/−^ mice. However, CCR4^−/−^ mice showed a marked decrease in CD11b+Ly6C^hi^ inflammatory macrophages, indicating that CCR4 signaling plays a crucial role in EAE by regulating the accumulation and effector function of inflammatory monocytes/macrophages [9].

## Invariant natural killer T cells

Invariant natural killer T (iNKT) cells are a specialized subset of T lymphocytes that bridge innate and adaptive immunity. They express a semi-invariant T-cell receptor (TCR) comprising a conserved α-chain (Vα14-Jα18 in mice, Vα24-Jα18 in humans) paired with a restricted set of β-chains. This TCR recognizes lipid antigens presented by CD1d molecule, a non-polymorphic MHC class I-like protein. iNKT cells are rapidly activated by lipid ligands such as α-galactosylceramide (α-GC) or through cytokine-mediated pathways involving IL-12, IL-18, and type I interferons from antigen-presenting cells. The specific nature of the activating ligand plays a crucial role in shaping the cytokine profile of iNKT cells; for instance, α-GC analogs with structural modifications can skew iNKT cell responses toward either Th1 or Th2 cytokine production, such as IFN-γ or IL-4, respectively [25–27].

In addition to their anti-inflammatory and regulatory roles, iNKT cells can exert inflammatory effects depending on the ligand and context. For example, certain ligands drive robust IFN-γ-dominated response, amplifying immune activation and contributing to inflammation. This functional plasticity allows iNKT cells to either resolve or exacerbate immune responses, depending on the balance of stimuli and the microenvironment. Their ability to rapidly produce large amounts of cytokines, including IFN-γ, TNF-α, IL-4, and IL-10, underscores their importance as key immunoregulators and their involvement in various pathological conditions, such as autoimmunity, cancer, and infections [25–27].

An investigation into monocyte differentiation dynamics during EAE progression has provided valuable insights into the roles and regulation of monocyte subsets, especially Ly6C^hi^ inflammatory monocytes, and their transition into M2 macrophages. Denney *et al*. reported that Ly6C^hi^ pro-inflammatory monocytes are among the earliest and most predominant cells to infiltrate the CNS during EAE. Upon recruitment, these monocytes differentiate into macrophages, that adopt either an M1 (inflammatory) or M2 (alternatively activated macrophage, AAM) phenotype. Research has revealed that the induction of iNKT cells strongly affects the fate of these monocytes. Stimulation of iNKT cells led to a reduction in the number of Ly6C^hi^ monocytes in the CNS and enhanced the proportion of M2 macrophages. This shift was associated with the improvement in neurological deficits in EAE, highlighting the therapeutic implications of modulating iNKT cell function. This conversion was shown to rely on an IL-4 and CD1d-dependent pathway, facilitated by activated iNKT cells, which supported the differentiation of Ly6C^hi^ monocytes into M2 macrophages. These findings suggest that modulating iNKT cell-mediated monocyte differentiation may represent a promising strategy for MS [10].

## Th17 cells and cytokine-driven monocyte modulation in neuroinflammation

Effector T cells, particularly T-helper 17 (Th17) cells, play a central role in immune responses by modulating monocyte behavior in both circulation and the CNS. These cells produce cytokines such as interleukin-17 (IL-17), interleukin-22 (IL-22), and GM-CSF, which significantly influence monocyte function and phenotype.

IL-17 can upregulate the expression of chemokine receptors like CCR2 on monocytes, a key factor in their migration to sites of inflammation. By increasing CCR2 expression, IL-17 facilitates the retention of inflammatory monocytes in circulation and their subsequent infiltration into inflamed tissues, including the CNS in MS [28]. Studies have demonstrated that IL-17-deficient mice exhibit reduced severity of EAE, with decreased infiltration of neutrophils and Ly6C^hi^ inflammatory monocytes into the CNS. This suggests that IL-17 is critical for recruiting these immune cells to the site of inflammation, thereby contributing to disease progression [29].

Th17 cells, through the secretion of IL-17 and GM-CSF, also significantly influence monocyte metabolism, promoting a pro-inflammatory state. Both IL-17 and GM-CSF enhance glycolysis in monocytes, leading to increased production of pro-inflammatory cytokines such as TNF-α, IL-1β, IL-6, and IL-12p70. This metabolic shift is facilitated by the upregulation of glucose transporters (GLUT-1, -3, and -4) and the transcription factor c-Myc, which are essential for the heightened glycolytic activity observed in inflammatory macrophages [30]. It has been observed that GM-CSF-deficient mice are resistant to EAE induction, highlighting the importance of GM-CSF in disease pathogenesis [31]. Conversely, during the resolution phase of inflammation or in anti-inflammatory environments orchestrated by regulatory T cells (Tregs), monocytes undergo metabolic reprogramming favoring oxidative phosphorylation (OXPHOS). This shift supports the differentiation of monocytes into non-classical patrolling phenotypes, which are associated with tissue repair and homeostasis. Tregs play a pivotal role in this process by modulating metabolic pathways to maintain immune tolerance and prevent excessive inflammation [32].

A study by Amorim *et al*. employed a combination of genetic fate mapping, gene targeting, and high-dimensional single-cell multiomics analyses to dissect the roles of IFNγ and GM-CSF in monocyte differentiation and function during EAE. They concluded that IFNγ and GM-CSF orchestrate complementary differentiation programs in monocytes during neuroinflammation. IFNγ drives the maturation of Ly6C^hi^ monocytes into inflammatory phagocytes, while GM-CSF licenses their effector functions [33].

Furthermore, King *et al*. investigated the dynamics and importance of CD11b^+^CD62L^-^Ly6C^hi^ monocytes in EAE-affected mice and, identifying a GM-CSF-dependent pathway responsible for mobilizing CD11b^+^CD62L^-^Ly6C^hi^ monocytes with colony-forming potential into the bloodstream prior to EAE relapses. This finding shows that recruitment and renewal of myeloid cells in the CNS are tightly controlled activities that precede the clinical onset of autoimmune demyelination. The study showed that circulating Ly6C^hi^ monocytes can cross the BBB, upregulate proinflammatory molecules, and differentiate into CNS dendritic cells and macrophages, thus emphasizing their ability to act as proinflammatory cells in the CNS, contributing to the pathology of EAE. Furthermore, the clinical symptoms of EAE manifest earlier and become more severe when Ly6C^hi^ monocytes abound in circulation, which underscores their central role in aggravating neuroinflammatory processes and worsening disease symptoms. This study focused on GM-CSF, which is critical for the egress of Ly6C^hi^ precursors from the bone marrow, and the survival of CNS myeloid cell populations during relapsing or chronic autoimmune demyelination [11]. Thus, targeting GM-CSF and its signaling pathways could be a promising therapeutic approach, enabling strategies directed at mobilization, migration, and differentiation of circulating Ly6C^hi^ monocytes to manage MS disease progression and symptom mitigation.

Interferon-gamma (IFNγ), alongside GM-CSF, plays a pivotal role in modulating monocyte functions during neuroinflammation, as evidenced in the EAE model [34, 35]. IFNγ promotes the maturation of Ly6C^hi^ monocytes into mature inflammatory phagocytes within the inflamed CNS or facilitates their differentiation into MHCII^+^ antigen-presenting cells at early stages. These MHCII^+^ cells are crucial for initiating T-cell responses thereby contributing to the autoimmune mechanism. Furthermore, this cytokine enhances the capacity of monocytes to process and present antigens, enabling the effective activation of T-cells and perpetuating neuroinflammatory processes.

## Janus kinase (JAK)/signal transducer and activator of transcription (STAT) pathway

As a brief introduction to additional research, the Janus kinase (JAK)/signal transducer and activator of transcription (STAT) signaling pathway is integral to immune cell proliferation, differentiation, and modulation in autoimmune neuroinflammation. In the context of EAE, and MS, the JAK/STAT pathway drives the activation and differentiation of key immune cells, such as T helper cells (Th1 and Th17) and monocytes, key players in cytokine-mediated inflammatory cascades.

By illuminating the importance of the pathway in EAE, Shuai Shao and coworkers concentrated on CCR2-dependent Ly6C^hi^ monocytes in EAE pathology, designated as major early CNS infiltrators, aggravating the neuroinflammatory environment. Monocytes inside the CNS become monocyte-derived dendritic cells, which are essential for antigen-presentation and amplification of the autoimmune response. The JAK/STAT5 axis is central to these processes, facilitating GM-CSF-induced recruitment and activation of inflammatory monocytes. This study demonstrated that JAK inhibitors mitigate GM-CSF signaling, attenuating monocyte differentiation, inflammatory cytokine production, and the maturation of dendritic cells, thereby reducing pathogenic T cells and EAE severity. These findings underscore the therapeutic potential of targeting AK/STAT signaling to alleviate neuroinflammation and tissue damage in MS [12].

On the other hand, type III interferon, IFN-λ, activates the JAK/STAT pathway through its receptor complex, which includes interferon lambda receptor 1 (IFNLR1) and interleukin-10 receptor 2 (IL-10R2), to regulate immune cells, thereby reducing inflammation and preventing an exaggerated immune response that can cause autoimmunity. Sherwani *et al*. showed that IFNLR1 deficiency in mice led to exacerbated EAE, indicating a regulatory role of IFN-λ in the severity of neuroinflammation. Monocytes and other myeloid cells appear to be affected by the modulation of this type. IFN-λ was shown to regulate neutrophil activation and migration into the CNS during early disease stages, as evidenced by increased neutrophil infiltration in the spinal cord of IFNLR1-deficient mice. Furthermore, IFNLR1-deficient macrophage cultures, displayed enhanced myelin peptide-reactive Th17 cell expansion without affecting Th1 cells, underscoring its selective modulation of macrophage-mediated T-cell responses. These data indicate IFN-λ as a prospective MS target because of its ability to modulate the immune response, which among other things, translates to the inhibition of monocytes and neutrophil activity [13].

## CX3CR1

Taking into account the acknowledged role of the operation of patrolling monocytes through CX3CR1, the study by Weihua Mai *et al*. found that mice deficient in CX3CR1 and subjected to EAE revealed increased disease manifestations compared to their wild-type counterparts with EAE. The exacerbation manifested as earlier disease onset, increased peak EAE scores, and heightened CNS pathology. The pathology was characterized by massive infiltration of inflammation, more severe demyelination, and drastic damage to Purkinje cells. Lack of CX3CR1 led to a noticeable accumulation of CD45^+^CD115^+^ CD11c^+^Ly6C^-^ dendritic cells within the brains of EAE mice, in line with the increased disease severity and pathological progression observed. These results highlight the central role of CX3CR1 in controlling the entry and operation of myeloid cells in the CNS. Furthermore, CX3CR1-deficient mice showed increased MHC-II expression on brain myeloid cells, suggesting that CX3CR1 regulates MHC-II expression in antigen-presenting cells (APCs), thereby influencing T-cell activation in the CNS. This regulation was linked to the overexpression of key MHC-II transcriptional regulators, Class II transactivator (CIITA), and interferon regulatory factor-1 (IRF-1), further highlighting the molecular pathways through which CX3CR1 modulates immune responses in EAE [14].

CX3CR1, primarily expressed by microglia and perivascular macrophages in the CNS, also plays a crucial role in monocyte function. This receptor regulates phagocytosis, inflammatory responses, and cell migration under both homeostatic and pathological conditions. Alterations in CX3CR1 expression or function in monocytes can significantly affect the behavior of resident macrophages in the CNS. Lampron *et al*. explored the role of CX3CR1 in microglial clearance of myelin debris during demyelination and remyelination using a cuprizone-induced model. Their findings revealed that CX3CR1 deficiency drastically impaired phagocytic capacity, leading to a marked accumulation of myelin debris in the corpus callosum. Microglia lacking CX3CR1 exhibited diminished endosomal activity, as evidenced by reduced internalization of myelin products and cholesterol crystals compared to wild-type. Although oligodendrocyte precursor cell (OPC) recruitment was comparable between CX3CR1-deficient and wild-type mice, the persistent myelin debris in CX3CR1-deficient mice disrupted efficient remyelination [36].

In contrast, the study by Ridderstad Wollberg *et al*. focused on the peripheral roles of CX3CR1 and explored the therapeutic potential of pharmacological inhibition of the receptor. Using AZD8797, a selective CX3CR1 inhibitor, they demonstrated that blocking CX3CR1 on peripheral leukocytes in a chronic-relapsing rat model of EAE significantly reduced disease severity. AZD8797 treatment led to decreased paralysis, reduced CNS inflammation, and improved axonal preservation by limiting the infiltration of CX3CR1-expressing immune cells into the CNS. Importantly, the inhibition was peripherally restricted, sparing the homeostatic functions of microglia and other CNS-resident macrophages. This study underscores the potential therapeutic benefit of selectively targeting CX3CR1 in the periphery to mitigate immune-driven damage in diseases like MS by reducing immune cell migration into the CNS [37].

## IL-10-producing regulatory B cells (Bregs)

Pennati *et al*. conducted a novel therapeutic study investigating the healing effects of IL-10-producing regulatory B cells (Bregs) in an EAE model. Transfusion of Bregs into mice with established EAE resulted in clinical recovery, accompanied by significant spinal cord remyelination. In Breg-treated mice, the CNS-resident CD11b+/CD45^int^Ly6C^–^ microglia and infiltrating CD11b^+^/CD45^high^ monocytes/macrophages were restored to a normalized state and polarized toward CD206^+^ M2-like macrophages, favoring a reparative and anti-inflammatory phenotype. The proliferation of neo-oligodendrocytes was also evident, as demonstrated by an increase in CNS cells with early oligodendrocyte progenitor cell differentiation markers (A2B5), premyelination, and more mature oligodendrocyte markers (GalC, O1, and MBP/MOG). This study emphasizes the therapeutic potential of Breg transfusion in the EAE model of MS, demonstrating its ability to normalize the CNS immune environment by reducing the pro-inflammatory myeloid cell function and promoting phenotypes conducive to CNS repair mechanisms [15].

## Acetylcholine-producing NK cells

Jiang *et al*. explored the role of acetylcholine (ACh)-producing natural killer (NK) cells in EAE, focusing particularly on the interaction dynamics between these NK cells and CCR2^+^Ly6C^hi^ monocytes/macrophages. The investigation revealed ChAT-positive NK cells, allowing these cells to produce ACh, especially under the inflammatory conditions observed in EAE. Such ChAT^+^ NK cells were characterized by several features that were not found in their ChAT- counterparts, including the cytotoxicity and capacity to secrete chemokines/cytokines. Intraventricular injection of ChAT+ NK cells into mice ameliorated CNS damage upon induction of EAE and reduced the entry of CCR2^+^Ly6C^hi^ monocytes into the CNS. This implies that ChAT+ NK cells directly neutralize these monocytes and inhibit the production of proinflammatory cytokines. It was shown in the research that ChAT^+^ NK cells and CCR2^+^Ly6C^hi^ monocytes form immunological synapses, activated through α7-nicotinic acetylcholine receptors (α7-nAChRs). This finding elucidates a potential route of the action of ChAT+ NK cells on monocytes/macrophages, thus, representing a new immune regulation mechanism [16].

## Monocytes and their derived antigen-presenting cells

Giladi *et al*. investigated the contribution of monocytes and their derivative antigen-presenting cells (APCs) to EAE in mice. Leveraging single-cell RNA sequencing to dissect the complexity and variability of monocyte subsets throughout the acute and chronic phases of the disease, they pinpointed eight unique monocyte subsets alongside three dendritic cell variations, each characterized by distinct transcriptional profiles hinting at diverse roles. A notable discovery was the identification of monocyte subsets expressing Cxcl10 and Saa3, marked by their pathogenic capacity within the CNS. Intriguingly, these pathogenic cells were not descendants of the circulating Ly6C^+^ monocytes, traditionally viewed as precursors to various monocyte-derived cell types, but instead originated from early myeloid cell progenitors. The investigation also highlighted differential infiltration patterns of monocyte subsets between the acute and chronic stages of EAE, with a pronounced increase in Cxcl10^+^ monocytes correlating with disease advancement. Specifically, the Cxcl10+ and Saa3+ monocyte subsets saw a significant reduction in the CNS following anti-CCR2 treatment, a shift that aligned with the alleviation of clinical symptoms. These insights underscore the therapeutic potential of targeting certain pathogenic monocyte subsets, including Cxcl10+ and Saa3+ monocytes, as a novel approach to MS management [17].

Moreover, Monaghan *et al*. delved into the gene expression landscapes of monocytes and APCs throughout the EAE course, uncovering distinct profiles for CNS-infiltrating monocytes and monocyte-derived APCs. The inflammatory milieu of the CNS markedly influenced monocyte gene expression, diverging from their differentiation trajectory within the CNS. These CNS-resident monocytes were found to express genes tied to proinflammatory cytokines and chemokines, a pattern that persisted as they evolved into APCs. The identification of Ccl17, Ccl22, and Ccr7 as distinguishing markers of monocyte-derived APCs from Ly6C^hi^ monocytes sheds light on the differentiation cues and functional capacities of these cells in CNS inflammation. This study presents evidence that the CNS environment during EAE prompts the activation and differentiation of monocytes, as reflected by shifts in gene expression relevant to cell activation, receptor signaling, and migration [18].

## Maturation of circulating classical monocytes in EAE

The study by Dagkonaki *et al*. sheds light on the critical process of monocyte maturation and its implications for EAE, serving as a pivotal exploration into the dynamics of monocyte functionality in autoimmune contexts, particularly MS. Central to immune responses, the CCR2^+^Ly6C^hi^ subset of monocytes, while initially immature in the bloodstream, can migrate to tissues upon specific signal reception. Here, they undergo a maturation or differentiation process into macrophages or dendritic cells, gaining capabilities crucial for immune modulation, such as pathogen phagocytosis, antigen presentation, and the production of cytokines and chemokines. The maturation of monocytes into antigen-presenting effector cells (MHCII^+^) with programmed death-ligand 1 (PD-L1) expression is essential for establishing peripheral immune tolerance and safeguarding against CNS autoimmunity.

Dagkonaki *et al*. have shown that treatment with oxidized mannan-conjugated myelin oligodendrocyte glycoprotein 35-55 (OM-MOG) effectively induces peripheral maturation of CCR2^+^Ly6C^hi^ monocytes into Ly6C^hi^MHCII^+^PD-L1^+^ cells. This maturation process plays a crucial role in reversing spinal cord inflammation and demyelination in MOG-induced EAE. Soluble OM-MOG, administered intradermally, was found to directly target the skin draining lymph nodes, engaging subcapsular sinus macrophages, and stimulating CCR2^+^Ly6C^hi^ monocytes to express MHC class II and PD-L1. This action blocks immune cell migration to the spinal cord, thereby reversing established lesions.

Further investigation using a neutralizing anti-PD-L1 antibody in vivo and dendritic cell-specific PD-L1 knockout mice revealed that PD-L1 expression on non-dendritic cells, including monocytes, is crucial for OM-MOG’s therapeutic impact. This underlines PD-L1’s significance in dampening autoimmune responses, with the activated monocytes showcasing markers indicative of their role in antigen presentation and immunosuppression. The efficacy of OM-MOG in preventing CNS-directed migration of these cells while retaining their rapid activation and presence in the periphery elucidates a novel immune tolerance mechanism, emphasizing the potential of targeting circulating CCR2^+^Ly6C^hi^ monocyte maturation as a therapeutic strategy for autoimmune CNS diseases like MS [19].

## Circadian rhythm and melatonin

In a recent study conducted by Ghareghani *et al.*, the role of circadian rhythms on Ly6C^low^ and Ly6C^hi^ monocytes, and particularly their expression of melatonin receptors before and after penetrating the brain, was investigated within a cuprizone-induced demyelination model. The study revealed that alterations in circadian rhythm, induced through shifts in light exposure, significantly affected melatonin and cortisol levels, thereby influencing monocyte function. Continuous light exposure reduced melatonin levels and elevated cortisol, enhancing the phagocytic activity of Ly6C^low^ patrolling monocytes while altering their infiltration patterns. In contrast, Ly6C^hi^ inflammatory monocytes displayed minimal circadian rhythm-dependent changes. Notably, continuous light exposure resulted in a marked increase in both the number and functionality of Ly6C^low^ monocytes, facilitating myelin debris clearance and promoting CNS recovery.

Chimeric mouse experiments using GFP-tagged donor mice revealed that brain-resident macrophages retained the expression of melatonin receptors MT1A and MT1B, whereas bone marrow-derived macrophages (Ly6C^low^ and Ly6C^hi^ monocytes) lost these receptor expressions upon migrating into the demyelinated brain environment. This finding suggests a dynamic control of melatonin receptor expression under the influence of the cell environment and their differentiation status. By giving detailed information about the behavior of monocytes in the context of MS, this study emphasizes the central role of circadian rhythms and hormonal interactions in modulating the activities of monocytes [20].

## Other hormonal regulation of monocyte dynamics in MS

In our review of the literature exploring hormones that influence the monocyte switch, no single hormone has been definitively identified as the primary driver of the transition from classical Ly6C^hi^ to patrolling Ly6C^low^ monocytes. However, several hormones have been implicated in modulating monocyte functions, particularly enhancing their phagocytic activity, phenotype, and cytokine secretion profiles, which are critical in the context of MS. Among these, glucocorticoids, estrogen, and testosterone emerge as key regulators.

Glucocorticoids (GCs), steroid hormones produced by the adrenal cortex, are integral to immune regulation, and stress adaptation through glucocorticoid receptor-mediated gene transcription. Synthetic GCs, such as methylprednisolone (MP), are widely employed during acute MS relapses for their potent anti-inflammatory and immunosuppressive properties [38–40]. Beyond their impact on T cells, Fischer *et al*. demonstrated that MP polarizes monocytes toward an anti-inflammatory M2-like phenotype. In MS patients, MP therapy upregulated anti-inflammatory markers like CD163 and IL-10 in monocytes, while suppressing pro-inflammatory markers such as IL1B. Notably, MP enhances monocyte migration toward CNS-associated chemokines like CCL2, facilitating recruitment to inflamed CNS sites, likely via intracellular signaling pathways such as focal adhesion kinase (FAK) rather than altered chemokine receptor expression [41]. This dual action of GCs-reducing inflammation and promoting reparative monocyte recruitment—underpins their therapeutic benefit in MS repair management.

Estrogen, particularly 17β-estradiol (E2), regulates monocyte dynamics through estrogen receptor (ER)-mediated signaling. Monocytes predominantly express ERα, and E2 modulates their activity by altering gene expression related to adhesion, chemotaxis, and cytokine production [42]. E2 reduces pro-inflammatory mediators like TNF-α and IL-1β while promoting anti-inflammatory markers like CD163 and IL-10, thereby fostering the development of alternative, anti-inflammatory monocytes. Furthermore, E2 upregulates CX3CR1, a key receptor for patrolling monocytes, potentially enhancing their vascular surveillance and anti-inflammatory functions. These effects suggest that estrogen contributes to the regulation of monocyte subsets, particularly in inflammatory and autoimmune contexts like MS [43].

Testosterone, the primary male sex hormone, also modulates immune responses, although its direct role in monocyte subset switching is less clear. Testosterone has been shown to suppress pro-inflammatory cytokine production, including TNF-α and IL-6, and may indirectly influence monocyte function by altering the immune microenvironment [44]. While studies on its effects on monocyte phagocytosis and migration have yielded inconclusive results [45], testosterone has been associated with increased T-cell apoptosis, which may indirectly affect monocyte behavior and immune regulation [46]. These findings suggest that while testosterone’s direct effects on monocyte transitions are limited, it still plays a role in modulating the inflammatory milieu in diseases like MS.

## Alternatively activated macrophages

Monocytes exhibit remarkable functional diversity upon recruitment to tissues, driven by their ability to differentiate into distinct macrophage subsets based on environmental cues. Ly6C^hi^ monocytes are predisposed to differentiate into pro-inflammatory M1 macrophages, which produce cytokines such as TNF-α and IL-6, amplifying inflammatory responses and contributing to tissue damage. In contrast, Ly6C^low^ monocytes are more likely to differentiate into anti-inflammatory M2 macrophages, which promote tissue repair and resolve inflammation. The differentiation pathways of these subsets are regulated by specific factors: Toll-like receptor (TLR) engagement and exposure to cytokines like IFN-γ and GM-CSF drive Ly6C^hi^ monocytes toward an M1 phenotype, while signals such as IL-4, IL-13, TGF-β, immune complexes, and A2AR agonists promote the polarization of Ly6C^low^ monocytes into M2 macrophages. Conversely, anti-inflammatory cytokines like IL-10 suppress M1 differentiation, while a persistently pro-inflammatory environment can hinder M2 polarization.

In the context of CNS injuries, M2 macrophages, also known as alternatively activated macrophages (AAMs), exhibit complex and dynamic roles. Evidence from the analysis of M1 and M2 marker expression on human monocyte-derived macrophages indicates that AAMs possess dual functionality in MS lesions. Although MS lesions predominantly express M1 markers, a subset of macrophages also display M2-associated markers, such as the mannose receptor (MR) and CD163, particularly in perivascular and foamy macrophages within active lesions. The M2-like phenotype is linked to anti-inflammatory and reparative functions, including myelin debris clearance, growth factor secretion, and modulation of the lesion microenvironment to promote remyelination. However, many macrophages in active MS lesions exhibit an intermediate activation state, co-expressing both M1 (pro-inflammatory) and M2 markers, reflecting the complexity of the in vivo lesion microenvironment. This intermediate state highlights the potential for therapeutic strategies aimed at shifting these macrophages toward a fully M2 phenotype, thereby enhancing their reparative and anti-inflammatory roles while mitigating their contributions to inflammation [47].

AAMs also facilitate remyelination by mitigating the inhibitory effects of extracellular matrix components [48]. One significant mechanism involves the degradation of chondroitin sulfate proteoglycans (CSPGs), which are major impediments to OPC recruitment and differentiation. CSPGs accumulate in demyelinated lesions and act as nonpermissive substrates, obstructing OPC adhesion, process outgrowth, and maturation into myelinating oligodendrocytes. Through the secretion of matrix metalloproteinases (MMPs), such as MMP-9, AAMs degrade the glycosaminoglycan chains of CSPGs, neutralizing their inhibitory effects. Experimental models, such as lysolecithin-induced demyelination, demonstrate that reducing CSPGs via enzymatic degradation or inhibition of their synthesis increases OPC numbers and enhances their differentiation within lesions, ultimately improving remyelination. By clearing CSPGs, AAMs transform the extracellular matrix into a permissive environment, enabling OPCs to extend processes and form compact myelin. This process exemplifies the critical regulatory role of AAMs in shifting the lesion microenvironment from one of inhibition to one that fosters regeneration and repair [48].

The research of Terrazas *et al*. focused on changes of the monocytes in the EAE model and emphasized the central role of Ly6C^hi^ monocytes and the development of AAMs during infection with *Taenia crassiceps* and its effects on EAE. The investigation showed that the infection of *T. crassiceps* instigates the differentiation of Ly6C^hi^ monocytes into PD-L2+ AAMs, which is manifested by expressing the alternative activation molecules such as the mannose receptor (MR; CD206) and programmed death-ligand 2 (PD-L2). CCR2 signaling emerged as a critical pathway for recruiting and differentiating Ly6C^hi^ monocytes into PD-L2+ AAMs, suggesting that helminth infection induces a directed monocyte activation cascade with potential therapeutic implications for EAE [21]. In addition, in adoptive transfer experiments where PD-L2^+^ AAMs originating from Ly6C^hi^ monocytes were introduced into EAE-induced mice, a significant decrease in disease incidence along with a delayed onset and reduction in clinical severity was detected. These findings underscore the potent immunosuppressive role of helminth-induced AAMs in modulating the autoimmune response, controlling inflammation, and minimizing tissue damage in EAE. By highlighting the differentiation of Ly6C^hi^ monocytes into PD-L2^+^ AAMs, this study offers insights into the therapeutic potential of helminth-induced immunomodulation for managing neuroinflammatory diseases like MS [21].

## Kinetics of classical monocytes in EAE model

One of the studies on TMEV virus-induced MS model investigated the role of CD11b^+^Ly6C^+^ monocytes, early exiting immature myeloid cells, comparing the weight of their disease progression. This study highlighted the importance of the innate immune response in governing the disease progression with the infiltrates in this phase being CD11b^+^Ly6C^+^ cells. They were characterized as cells with suppressive activity against responses of CD4^+^ and CD8^+^ T cells, focusing attention on their role in the progression of demyelinating diseases. Depletion of CD11b^+^Ly6C^+^ cells in TMEV-infected mice led to a significant reduction in disease severity, lower myelin-specific CD4^+^ T cell responses, and a milder inflammatory immune response in the CNS. Interestingly, this depletion also enhanced virus-specific T-cell responses with higher levels of IFN-γ and IL-17 and lower levels of IL-10 in the CNS, indicating that CD11b^+^Ly6C^+^ cells may function as myeloid-derived suppressor cells, influencing the overall immune response and contributing to disease development [22].

In another study, Mishra *et al*. analyzed the kinetics of CD11b^+^CCR2^+^Ly6C^hi^ proinflammatory monocytes in an EAE model and observed a marked and sustained elevation of these monocytes in blood from the second day posts-EAE induction, peaking at day 3 and remaining elevated even until clinical symptoms onset. The blood levels of these monocytes decreased to baseline levels at clinical onset, while their invasion of the spinal cord correlated with the peak of the disease. At disease onset, the levels of monocytes in the blood were inversely related to their presence in the spinal cord, highlighting their role in disease progression. In addition, laquinimod treatment, which prevented EAE onset, was associated with prolonged presence of proinflammatory monocytes in the blood, along with reduced levels of CD62L and MMP-9, CCR2 and CCL2 in the spinal cord—key factors involved in monocyte chemotaxis. This study emphasized the function of the JAK/STAT signaling pathway in modulating monocyte migration and suggests that laquinimod’s effects on this pathway offer a protective mechanism in the EAE model, which could provide therapeutic strategies for MS treatment [23].

Helmersson *et al*. investigated the effects of paquinimod, a quinoline-3-carboxamide compound, in the EAE model, demonstrating that early treatment post-EAE induction reduced disease severity. This reduction was linked to decreased priming of antigen-specific CD4+ T cells and a lower frequency of IFN-γ and IL-17 cells in draining lymph nodes, without suppressing T-cell division. The study emphasized the pivotal role of Ly6C^hi^ inflammatory monocytes in early EAE phases. Both paquinimod treatment and anti-Gr-1 antibody administration during the induction phase significantly ameliorated disease severity. However, the anti-Gr-1 antibody depletes not only Ly6C^hi^ monocytes but also Ly6G+ neutrophils, indicating that its effects are not specific to monocytes. Additionally, paquinimod treatment reduced CD11b^+^ cell accumulation, including CD11b^+^Ly6C^hi^ inflammatory monocytes and CD11b^+^CD11c^+^ dendritic cells in the spleen, though not in the bone marrow or draining lymph nodes [24]. These findings support the potential of targeting Ly6C^hi^ inflammatory monocytes and related immune cells as a therapeutic approach for MS, while also highlighting the broader effects of anti-Gr-1 treatment on immune cell populations

## Monocyte switch as a therapeutic target in Alzheimer’s disease and MS

Modulating innate immunity through NOD2 signaling offers a promising therapeutic avenue for Alzheimer’s disease (AD), leveraging similar mechanisms to those explored in MS. MDP, a NOD2 ligand, has been shown to drive the transition of classical Ly6C^hi^ monocytes to non-classical Ly6C^low^ patrolling monocytes, a shift that mitigates neuroinflammation and addresses pathological hallmarks of neurodegenerative diseases. In AD models, research by Rivest’s group [5, 7, 49] demonstrated that patrolling monocytes contribute to amyloid clearance and inflammatory regulation. Specifically, MDP administration in APPswe/PS1 mouse models delayed cognitive decline by enhancing amyloid clearance through upregulation of phagocytic markers such as TREM2, CD68, and LAMP2. Additionally, NOD2 activation bolstered BBB integrity by increasing the expression of transporters like LRP1 and ABCB1, facilitating the removal of amyloid from the CNS via the “sink effect” [50].

These mechanisms contrast with MS, where the NOD2-mediated monocyte switch primarily resolves inflammation and supports myelin repair. While both conditions benefit from monocyte modulation, AD therapies prioritize amyloid clearance and BBB stabilization, whereas MS interventions focus on mitigating immune-mediated demyelination. Notably, the effects of MDP in AD display sex-specific variations, with males exhibiting enhanced peripheral amyloid clearance and females showing CNS-localized benefits. These findings highlight the broader potential of monocyte-targeted therapies in neurodegenerative diseases, advocating for tailored strategies that address disease-specific mechanisms and individual patient profiles.

Future studies should leverage advanced tools like the Ms4a3-reporter mouse model [51], which enables more precise monocyte-fate mapping compared to CX3CR1-reporter mice. This model, alongside single-cell RNA sequencing and spatial transcriptomics, can uncover monocyte heterogeneity and plasticity in MS. Investigating how established MS therapies, such as interferons, dimethyl fumarate, and fingolimod, influence the monocyte switch from Ly6C^hi^ to Ly6C^low^ subsets, either directly or indirectly, would also be valuable. These insights could optimize current treatments and guide the development of targeted therapies.

## Conclusion

The dynamic switch between monocyte subsets and their modulation of infiltration into the CNS underscore their pivotal, yet complex, role in the pathophysiology and potential therapeutic targeting of MS. This review highlighted how the transition from pro-inflammatory to reparative monocyte functions can beneficially impact MS progression, pointing toward a nuanced therapeutic opportunity to harness their plasticity for disease management. Despite the growing evidence supporting monocyte targeting in MS therapy, our current understanding of their specific functions and involvement in MS remains incomplete. This review paper shines a light on the most relevant studies using MS models to investigate monocyte dynamics, emphasizing the need for further detailed investigations. These studies, while advancing our knowledge, also underscore the intricate balance monocytes maintain between exacerbating and alleviating disease, thus advocating for a deeper exploration into monocyte-mediated mechanisms to fully exploit their therapeutic potential in MS.

## Data Availability

There are no new data associated with this article.
